# Development of a predictive tool for long-term prognosis in clear cell adenocarcinoma of the cervix: a large population-based real-world study

**DOI:** 10.3389/fmed.2025.1606685

**Published:** 2025-06-20

**Authors:** Yanhong Wang, Yi Ouyang, Yiping Chen, Zhigang Bai, Xinping Cao, Qunrong Cai, Qin Xu

**Affiliations:** ^1^Department of Radiation Oncology, The Second Affiliated Hospital of Fujian Medical University, Quanzhou, Fujian, China; ^2^Clinical Oncology School of Fujian Medical University, Fujian Cancer Hospital, Fuzhou, Fujian, China; ^3^The Graduate School of Fujian Medical University, Fuzhou, Fujian, China; ^4^Department of Radiotherapy, State Key Laboratory of Oncology in South China, Sun Yat-sen University Cancer Center, Collaborative Innovation Center for Cancer Medicine, Guangzhou, Guangdong, China

**Keywords:** clear cell adenocarcinoma of the cervix, nomogram, OS, FIGO stage, risk stratification

## Abstract

**Background:**

Clear cell adenocarcinoma of the cervix (CCAC) is a rare malignancy without a well-established prognostic model. Our study aimed to develop and validate a nomogram to estimate overall survival in CCAC patients.

**Methods:**

We collected data on 630 CCAC patients from the Surveillance, Epidemiology, and End Results (SEER) database (2000–2021). Missing clinicopathological data were imputed using the missForest package. The imputed dataset served as the training cohort, while the dataset with missing values removed acted as the validation cohort. The nomogram’s performance was assessed through discriminative ability, calibration, C-index, AUC, and calibration plots. Clinical benefits were compared against the International Federation of Gynecology and Obstetrics (FIGO) staging using decision curve analysis (DCA), net reclassification index (NRI), and integrated discrimination improvement (IDI).

**Results:**

The nomogram, based on nine variables, demonstrated strong discriminative power, with C-index values of 0.82 for the training cohort and 0.81 for the validation cohort, and AUCs exceeding 0.80 in both sets. Calibration plots showed a strong agreement between the nomogram’s predictions and actual outcomes in both cohorts. The NRI values for the training set were 0.21 for 3-year, 0.20 for 5-year, and 0.30 for 10-year overall survival (OS) predictions, and for the validation set were 0.34 for 3-year, 0.25 for 5-year, and 0.31 for 10-year OS predictions. The IDI results for the training set were 0.17 across 3-, 5-, and 10-year OS predictions, and for the validation set were 0.21 for 3-year, 0.17 for 5-year, and 0.15 for 10-year OS predictions. The nomogram significantly outperformed the FIGO criteria (*p* < 0.01), and DCA highlighted its superior clinical utility in identifying high-risk patients.

**Conclusion:**

The nomogram, which integrates treatment data, was successfully developed and validated to assist clinicians in assessing the prognosis of CCAC patients. It demonstrated superior performance to FIGO criteria in predicting overall survival.

## Introduction

Clear cell adenocarcinoma of the cervix (CCAC) is a rare and aggressive subtype of cervical cancer, representing approximately 4–9% of all cervical adenocarcinomas (1). The majority of patients with CCAC have been reported to have exposure to diethylstilbestrol (DES) (2, 3). The information regarding the clinical behavior, pathology, and prognosis of these tumors is limited and inconsistent due to their rarity. At present, prognostic evidence for CCAC is still based on retrospective studies with a small patient sample within single institution (1, 4–9).

Consequently, there are no specific recommendations for predicting the outcome or managing CCAC. Instead, clinicians typically use the same approaches that are used for more common types of cancer, such as squamous cell carcinoma (SCC), typical adenocarcinoma (ACC), and adeno-squamous carcinoma (ASC). Multiple studies have revealed that patients with CCAC usually have a poorer prognosis than those with SCC or ACC (1). These results highlight the need for an independent evaluation of CCAC. Regrettably, we lack a specific model to predict the prognosis of CCAC patients.

We commonly use the International Federation of Gynecology and Obstetrics (FIGO) staging system to assess the prognosis of gynecological patients. However, this staging system has limitations such as low precision, ignoring other significant factors (such as age, tumor grade, and histologic type), and poor performance in predicting survival on a personal level (10–13). Hence, a more personalized model is essential for predicting prognosis for patients with CCAC.

Nomograms have been commonly used to predict the prognosis of cancer in recent years (14). They estimate the individual probability of specific clinical outcomes by taking into account various prognostic variables and aligning with the goals of personalized medicine (14). In the current study, we sought to develop a nomogram to forecast the prognosis of CCAC patients by utilizing a large CCAC cohort from the Surveillance, Epidemiology, and End Results (SEER) database.

## Methods

### Registration and ethics approval

This study has been registered at the Chinese Clinical Trial Registry (registration ID: ChiCTR2500097240, https://www.chictr.org.cn/showproj.html?proj=252957) and approved by the Ethics Committee of the Second Affiliated Hospital of Fujian Medical University (Ethics Approval No. [2024] (708)).

### Patient selection

The Surveillance, Epidemiology, and End Results (SEER) data analyzed in this study were retrieved in 08/2024, before access restrictions were imposed on Chinese researchers. We conducted all research in compliance with the SEER Data Use Agreement (DUA) at the time of data download. The SEER database contains cases solely from the United States, and female patients diagnosed with CCAC between 2001 and 2021 were enrolled through SEER*Stat 8.4.3. The eligibility criteria were defined as follows: (i) the International Classification of Diseases (ICD) code 0–3 morphology 8310/3; (ii) site record of ICD-O-3 encompassed cervix uteri; (iii) active follow-up to guarantee dependable patient status. The exclusion criteria were as follows: (i) patient died within 1 month or was followed up less than 1 month since initial diagnosis.

### Variable definition

In our study, we take into account 10 clinicopathological factors from SEER database, including age at diagnosis, race, marital status at diagnosis, tumor size, tumor grade, FIGO criteria-based tumor stage, surgery pattern of primary site, lymphadenectomy, radiation, and chemotherapy. The types of surgery performed at the primary cancer site were categorized as follows: none, total hysterectomy, radical hysterectomy, local surgery, exenteration, hysterectomy, or other. FIGO criteria-based tumor stage was classified according to the SEER Combined Summary Stage (2004+) as localized (including FIGO 2018 stages IA and IB), regional (including FIGO 2018 stages IIA, IIB, IIIA, IIIB, and IIIC), and distant (including FIGO 2018 stages IVA and IVB) (15).

### Statistical analysis

In this study, we found that some data were missing for all factors except radiation and chemotherapy. Since we only retrospectively identified 630 patients with CCAC from the SEER database, and selection bias may occur if we removed patients with incomplete data, we imputed all missing data to make use of all available patient records. To fill missing data, we used the missForest method (implemented in the R statistical environment using missForest package) that can deal with missing values in both continuous and categorical variables (16). Then, the database after imputation was treated as training cohort, and the database with missing value deletion was treated as validation cohort.

In this study, the primary endpoint was overall survival (OS), defined as the period extending from the date of diagnosis to the date of death from any cause or to the date of the final follow-up, which was conducted in November 2021. Data from patients who survived to the final follow-up were right-censored. Univariate Cox proportional hazards regression analyses were conducted on all 10 factors. Factors with a *p*-value of less than 0.05 in both the univariate and multivariate models were recognized as independent prognostic factors. While age was initially modeled as a continuous linear variable, we formally tested for non-linearity using restricted cubic splines with three knots. The significance of non-linear terms was tested via analysis of variance (ANOVA) of the Cox model (17). A stepwise regression analysis based on the Akaike information criterion (AIC) was employed to select variables for the development of the nomogram (14). The nomogram was used to estimate the probabilities of OS at 3-, 5-, and 10-years post-diagnosis. In the present study, a total of 306 events were recorded, which aligns with Harrell’s recommendation that the number of events should be at least 10 times greater than the number of covariates (14). The degree of multicollinearity among covariables in the nomogram was evaluated by calculating the variance inflation factor (VIF). Covariables with a VIF value exceeding 4.0 were not included in the final mode (18).

The nomogram’s predictive accuracy was assessed through the concordance index (C-index) calculated by bootstrapping and area under receiver operating characteristics curve (AUC), and calibrating performance was evaluated using the calibration curve which was calculated by bootstrapping. The C-index and AUC values range between 0.5 and 1.0, with 0.5 representing random prediction and 1.0 indicating perfect discrimination. Generally, C-index and AUC values exceeding 0.75 indicate a satisfactory predictive performance. The nomogram’s clinical utility relative to FIGO criteria-based tumor stage was assessed using the net reclassification index (NRI) (19, 20), integrated discrimination improvement (IDI) (20), and decision curve analysis (DCA) (21). The X-tile software was employed to determine the optimal cutoff point for risk stratifications based on the total points derived from the nomogram, and all patients were categorized into three risk groups: low risk, median risk, and high risk (22). The Kaplan–Meier method was used to compare the overall survival among the three risk groups defined by the nomogram with those stratified by the FIGO criteria-based tumor stage. The C-index, AUC, calibration curve, NRI, IDI, DCA curve, and risk stratification were conducted in both the training and validation cohorts. All *p*-values presented are derived from two-sided tests with a significance level of *α* = 0.05. The statistical analyses were conducted using R version 4.3.3.^1^

In accordance with the journal’s guidelines, we will provide our data for independent analysis by a selected team by the Editorial Team for the purposes of additional data analysis or for the reproducibility of this study in other centers if such is requested.

## Results

Between 2000 and 2021, a total of 630 women were diagnosed with CCAC. The median duration of follow-up for the entire cohort was 37.5 months, with an interquartile range (IQR) of 14.25–109.75 months. It is noteworthy that approximately 50% of these patients eventually died. The clinicopathological characteristics of the training cohort (after imputation) and the validation cohort (before imputation) are detailed in Table 1. The median age of the patients was 59 years (IQR: 45–71 years). The majority of the CCAC patients were of white ethnicity, accounting for 76.98% of the cohort. Over half of the patients presented with tumors exceeding 2 cm in size, exhibited G3/4 tumor differentiation, or were diagnosed at a regional or distant stage. Notably, the rate of lymph node involvement was as high as 26.83%, a finding consistent with other studies (5, 23). The principal surgical intervention for CCAC patients included total hysterectomy and radical hysterectomy. The incidence of patients receiving radiotherapy and chemotherapy was 61.27 and 50.32%, respectively.

**Table 1:** Clinical characteristics of patients with CACC.

Characteristics	Training cohort [*n* = 630, cases (%)]	Validation cohort [*n* = 247, cases (%)]	*p*-value
Age (median, IQR)	59 (45,71)	60 (47, 72)	0.5798
Race			0.6752
White	488 (77.46%)	197 (79.76%)	
Black	74 (11.75%)	24 (9.72%)	
Asian/Alaska Indian	68 (10.79%)	26 (10.53%)	
Marital status			0.8059
Single/unmarried	176 (27.94%)	69 (27.94%)	
Married	280 (44.44%)	117 (47.37%)	
Divorced/separated	62 (9.84%)	23 (9.31%)	
Widowed	112 (17.78%)	38 (15.38%)	
Tumor size			0.0762
< 2 cm	114 (18.10%)	60 (24.29%)	
2.0-4 cm	176 (27.94%)	71 (28.74%)	
≥ 4.0 cm	340 (53.94%)	116 (42.34%)	
Grade			0.2116
1	21 (3.33%)	9 (3.64%)	
2	66 (10.48%)	33 (13.36%)	
3	451 (71.59%)	159 (64.37%)	
4	92 (14.60%)	46 (18.62%)	
Lymph node metastasis			0.7222
No	433 (68.73%)	166 (67.21%)	
Yes	197 (31.27%)	81 (32.79%)	
Tumor stage^a^			0.2318
Local	292 (46.35%)	128 (51.82%)	
Regional	232 (36.83%)	87 (35.22%)	
Distant	106 (16.83%)	32 (12.96%)	
Primary site surgery			<0.001
No	231 (36.67%)	56 (22.67%)	
L/E/Hys/other	81 (12.86%)	25 (10.12%)	
Total hysterectomy	159 (25.24%)	77 (31.17%)	
Radical hysterectomy	159 (25.24%)	89 (36.03%)	
Lymphadenectomy			<0.001
No	302 (47.94%)	80 (32.38%)	
Yes	328 (52.06%)	167 (67.61%)	
Radiation			1
No	244 (38.73%)	96 (388.87%)	
Yes	386 (61.27%)	151 (61.13%)	
Chemotherapy			0.7446
No	313 (49.68%)	119 (48.18%)	
Yes	317 (50.32%)	128 (51.82%)	
Status			0.0118
Alive	324 (51.43%)	151 (61.13%)	
Dead	306 (48.57%)	96 (38.87%)	
Survival months (median, IQR)	37.50 (14.25, 109.75)	48 (18, 108.50)	0.7845

CCAC, clear cell adenocarcinoma of the cervix; IQR, interquartile range; L/E/Hys/other, local surgery, exenteration, and other surgery types.

^a^Tumor stage was determined using the International Federation of Gynecology and Obstetrics (FIGO) criteria.

As shown in Table 2, age, marital status, tumor stage, primary site surgery, and chemotherapy were identified as independent prognostic factors for CCAC. Multivariate Cox regression revealed that older age, divorced/separated marital status, regional/distant disease stage, and lack of either surgery or chemotherapy were all independently associated with poorer prognosis. The stepwise regression analysis revealed that a model incorporating variables including age, marital status, tumor size, lymph node metastasis, tumor stage, primary site surgery, lymphadenectomy, radiation, and chemotherapy demonstrated the lowest AIC value within the training set. Furthermore, all variable inflation factor (VIF) values were below 4, indicating the absence of multicollinearity among the selected variables.

**Table 2:** Univariate and multivariate Cox analyses on variables for the prediction of overall survival of CCAC patients.

Variable	Univariate analysis			Multivariate analysis		
	HR	95%CI	*p*-value	HR	95%CI	*p*-value
Age (years)	1.03	1.02–1.04	<0.001	1.03	1.02–1.04	<0.001
Race						
White	1			1		
Black	1.43	1.02–1.99	0.036	1.25	0.87–1.79	0.227
Asian/Alaska Indian	0.79	0.54–1.17	0.240	0.94	0.63–1.40	0.747
Marital status						
Single/unmarried	1			1		
Married	1.13	0.84–1.52	0.422	0.84	0.62–1.15	0.275
Divorced/separated	2.08	1.40–3.11	<0.001	1.56	1.02–2.37	0.039
Widowed	2.25	1.61–3.13	<0.001	0.90	0.6–1.35	0.613
Tumor size						
< 2 cm	1			1		
2.0–4 cm	1.40	0.92–2.11	0.116	1.28	0.83–1.99	0.265
≥ 4.0 cm	3.38	2.34–4.86	<0.001	1.54	0.99–2.39	0.056
Grade						
1	1			1		
2	2.58	0.90–7.40	0.077	2.24	0.77–6.50	0.138
3	4.27	1.58–11.49	0.004	1.96	0.71–5.39	0.194
4	5.40	1.96–14.89	0.001	2.67	0.94–7.61	0.065
Lymph node metastasis						
No	1			1		
Yes	2.74	2.18–3.44	<0.001	1.35	0.99–1.84	0.056
Tumor stage^a^						
Local	1			1		
Regional	3.12	2.37–4.11	<0.001	2.62	1.81–3.79	<0.001
Distant	8.19	6.00–11.18	<0.001	5.11	3.20–8.18	<0.001
Primary site surgery						
No	1			1		
L/E/Hys/other	0.34	0.23–0.48	<0.001	0.67	0.43–1.06	0.091
Total hysterectomy	0.26	0.19–0.35	<0.001	0.48	0.30–0.77	0.002
Radical hysterectomy	0.18	0.13–0.25	<0.001	0.35	0.20–0.59	<0.001
Lymphadenectomy						
No	1			1		
Yes	0.28	0.22–0.35	<0.001	0.72	0.48–1.10	0.133
Radiation						
No	1			1		
Yes	1.54	1.21–1.97	0.001	0.77	0.57–1.03	0.082
Chemotherapy						
No	1			1		
Yes	1.36	1.08–1.71	0.007	0.60	0.45–0.80	<0.001

CCAC, clear cell adenocarcinoma of the cervix; CI, confidence interval; HR: hazard ratio; L/E/Hys/other, local surgery, exenteration, and other surgery types.

^a^Tumor stage was determined using the International Federation of Gynecology and Obstetrics (FIGO) criteria.

**Figure 1:**
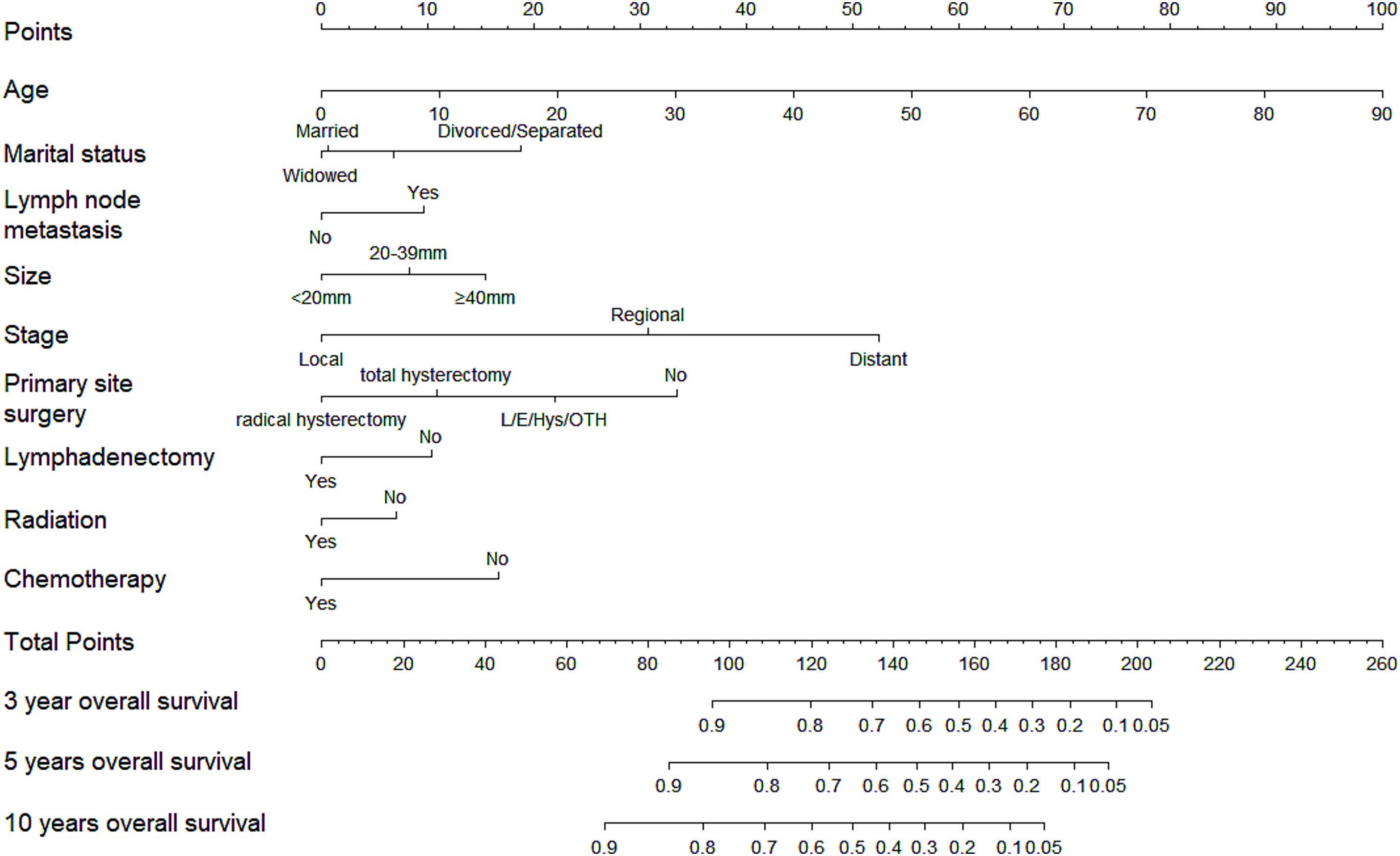
Nomogram of 3-, 5-, and 10-year overall survival for patients with clear cell adenocarcinoma of cervix (CCAC).

A nomogram incorporating the aforementioned nine variables was developed for CCAC, as shown in Figure 1. The nomogram assigned the highest weight to age as a linear predictor (HR = 1.03 per year, 95% confidence interval (CI) 1.02–1.04). Formal testing revealed no evidence of non-linearity (*p* = 0.48). The C-index value was 0.82 (95% confidence interval (CI), 0.79–0.84) for the training cohort and 0.81 (95%CI, 0.77–0.84) for the validation cohort, as detailed in Table 3. The AUC values were consistently high, with 0.87 observed for the prediction of 3-year, 5-year, and 10-year OS in the training cohort. In the validation cohort, the AUC values were 0.85, 0.84, and 0.81 for the prediction of 3-year, 5-year, and 10-year OS, respectively, as shown in Supplementary Figure 1. Supplementary Figure 2 illustrates that the calibration curves of the nomogram closely align with the diagonal dotted line for both the training and validation cohorts, indicating a high degree of concordance between the predicted and observed survival probabilities. In conclusion, the nomogram for CCAC exhibited significant discriminative and calibration capabilities.

**Table 3:** NRI, IDI, and C-index of the nomogram and FIGO criteria-based tumor staging alone in survival prediction for CCAC patients.

Index	Training cohort			Validation cohort		
	Estimate	95%CI	*p*-value	Estimate	95%CI	*p*-value
C-index						
The nomogram	0.82	0.79–0.84		0.81	0.77–0.84	
The FIGO criteria-based tumor staging	0.72	0.69–0.74		0.73	0.69–0.77	
Change	0.10	0.07–0.12	<0.001	0.08	0.05–0.11	<0.001
NRI (vs. the FIGO criteria-based tumor staging)						
For 3-year OS	0.21	0.09–0.33		0.34	0.18–0.53	
For 5-year OS	0.20	0.09–0.31		0.25	0.10–0.39	
For 10-year OS	0.30	0.21–0.42		0.31	0.14–0.47	
IDI (vs. the FIGO criteria-based tumor staging)						
For 3-year OS	0.17	0.11–0.23	<0.001	0.21	0.13–0.33	<0.001
For 5-year OS	0.17	0.12–0.23	<0.001	0.17	0.10–0.27	<0.001
For 10-year OS	0.17	0.12–0.22	<0.001	0.15	0.08–0.26	<0.001

CCAC, clear cell adenocarcinoma of the cervix; CI, confidence interval; FIGO, the International Federation of Gynecology and Obstetrics; HR, hazard ratio; IDI, integrated discrimination improvement; NRI, net reclassification index.

The comparative analysis of the nomogram’s precision against the FIGO criteria-based tumor staging was conducted using changes in the C-index, NRI, and IDI, as presented in Table 3. The finding indicated that the nomogram provided superior prognostic accuracy over the FIGO criteria-based tumor staging. The C-index improvement in the training and validation cohort was 0.10 (95%CI, 0.07–0.12) and 0.08 (95%CI, 0.05–0.11), respectively. The NRI values for the 3-, 5-, and 10-year OS in the training cohort were 0.21 (95%CI, 0.09–0.33), 0.20 (95%CI, 0.09–0.31), and 0.30 (95%CI, 0.21–0.42), respectively. In the validation cohort, these values were 0.34 (95%CI, 0.18–0.53), 0.25 (95%CI, 0.10–0.39), and 0.31 (95%CI, 0.14–0.47). The IDI values for 3-, 5-, and 10-year OS were 0.17 (95%CI, 0.11–0.23), 0.17 (95%CI, 0.12–0.23), and 0.17 (95%CI, 0.12–0.22) in the training cohort, and these values in the validation cohort were 0.21 (95%CI, 0.13–0.33), 0.17 (95%CI, 0.10–0.27), and 0.15 (95%CI, 0.08–0.26).

The DCA curve was employed to assess the clinical benefits of the nomogram in comparison with the FIGO criteria-based tumor staging. The DCA curves demonstrated that the nomogram provided a greater net benefit compared to the FIGO criteria-based tumor staging across nearly all threshold probabilities for both the training and validation cohorts. This suggests that the nomogram is more effective in predicting 3-, 5-, and 10-year OS, as shown in Supplementary Figure 3.

**Figure 2:**
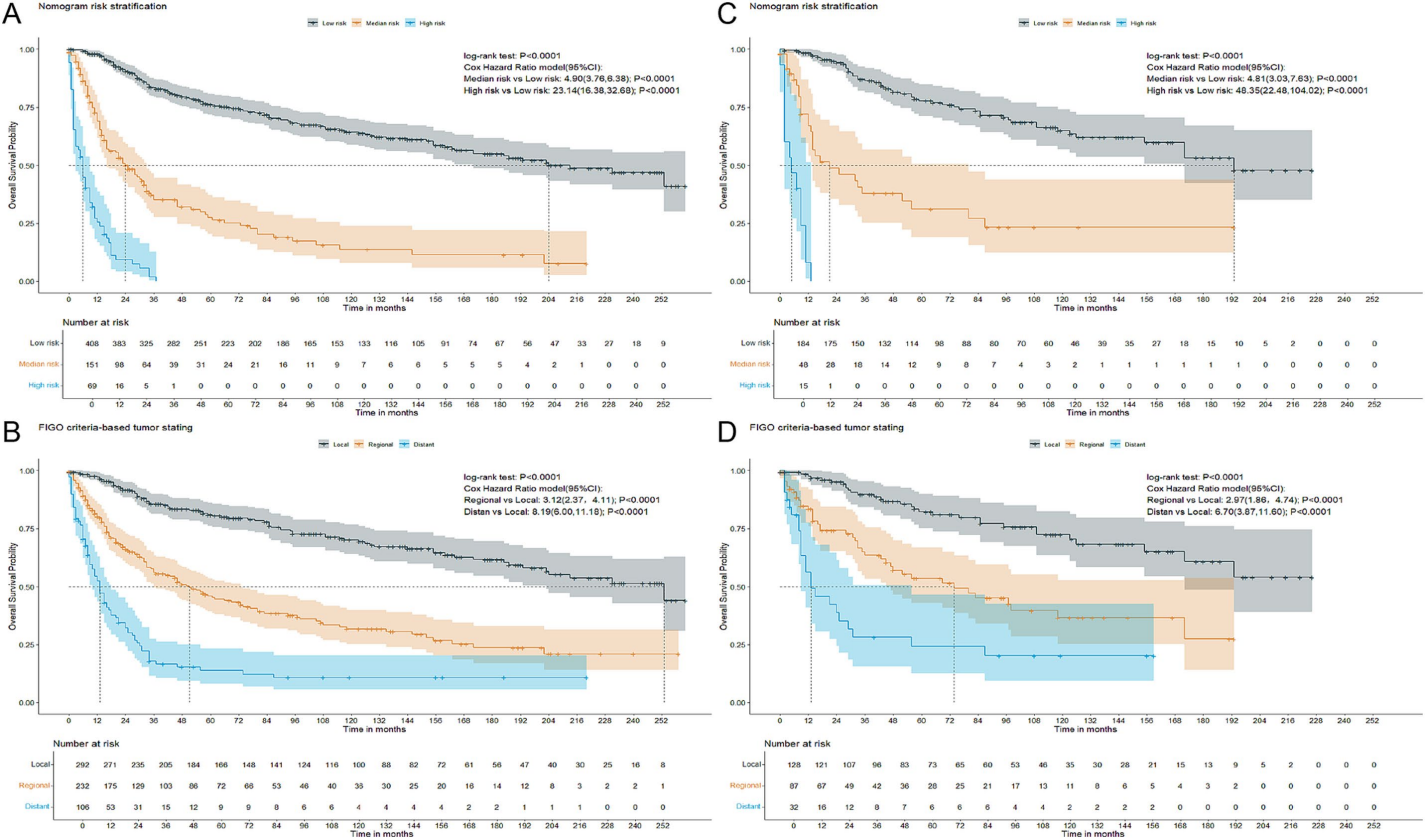
Kaplan–Meier overall survival curves of patients with CCAC at different FIGO criteria-based tumor stages or with different risks stratified by the nomogram. **(A)** CCAC patients in the training cohort at different risks stratified according to the nomogram. **(B)** CCAC patients in the training cohort at different stages classified according to the FIGO criteria-based tumor staging. **(C)** CCAC patients in the validation cohort at different risks stratified according to the nomogram. **(D)** CCAC patients in the validation cohort at different stages classified according to the FIGO criteria-based tumor staging. CCAC, clear cell adenocarcinoma of the cervix; FIGO, The International Federation of Gynecology and Obstetrics.

Ultimately, patients were stratified into three risk groups based on the total points calculated by the nomogram: low risk (total point≤150.67), median risk (151.35 ≤ total point ≤191.32), and high risk (total point ≥191.67). The Kaplan–Meier OS curves revealed a more distinct separation among the three risk groups compared to the differentiation observed with the FIGO criteria-based tumor staging in both training and validation cohorts, as illustrated in Figure 2.

## Discussion

CCAC is a rare malignant neoplasm, with limited clinical data and studies available to assess its prognostic outcomes. To address this gap, we developed a nomogram to predict the prognosis for individuals diagnosed with CCAC. Utilizing stepwise regression guided by the minimal AIC criterion, we identified and incorporated nine variables into the nomogram’s construction. The validation process confirmed that the nomogram exhibits excellent discriminative and calibration capabilities. Moreover, it provides a more precise prediction of prognosis compared to FIGO criteria-based tumor staging, effectively differentiating risk groups, so as to promote the follow-up plan and individualized treatment of this tumor.

Earlier research on CCAC has implicated certain factors that may impact OS, such as lymph node metastasis. This factor was also thoroughly taken into consideration in the current research. Yang et al. (24), Li et al. (25), and Thomas et al. (23) have suggested that lymph node metastasis negatively affects OS. However, our results indicate that lymph node metastasis is not an independent predictor for OS. It is important to note that lymphadenectomy was performed in only 43.97% of the patients in the present study, which implies that some metastatic lymph nodes may have been undetected, potentially leading to false-negative conclusions. While lymphadenectomy may hold prognostic value, our study did not find it to confer a therapeutic benefit. Contrasting with Hanselaar et al. results in their 55-patient cohort (26), tumor grade showed no independent prognostic value in our study (*n* = 630), a discrepancy that may be attributed to our substantially larger sample size (11.5 times greater), which reduces random error and increases the reliability of negative findings. This observation aligns with the statistical principle that small sample sizes are prone to overestimate effect sizes (27). Our study also recognized marital status as a prognostic factor using a nomogram, revealing that divorced or separated individuals faced a higher risk of mortality. To date, no other investigation has established a link between marital status and the survival rates of CCAC patients.

The role of radiation therapy has also been a subject of debate. Many retrospective studies have not found radiation to be independently associated with OS (6, 8, 24). However, a retrospective population-based cohort retrospective study suggested that the combination of external beam radiotherapy and brachytherapy could improve OS (25). In our study, radiation was not identified as an independent factor for improving OS. Nevertheless, incorporating radiation into the nomogram reduced the AIC value, highlighting its significance in forecasting OS for CCAC patients.

Clear cell adenocarcinoma of the cervix (CCAC) is recognized as a special subtype of adenocarcinoma of the cervix (ACC), which is associated with poorer outcomes and shows resistance to both radiotherapy and chemotherapy when compared to squamous cell carcinoma (SCC) (28, 29). Consequently, a consensus among researchers advocates for surgery as the primary treatment modality for ACC, including those cases presenting with locally advanced stages (30, 31). Our study emphasizes the critical role of surgical intervention, particularly total hysterectomy and radical hysterectomy, in the management of CCAC. The significant survival benefit of surgery in CCAC patients was further confirmed by Li et al. (25). It is important to note that more extensive tumor resections, such as total hysterectomy and radical hysterectomy, rather than local tumor destruction or single hysterectomy, may offer a protective effect in CCAC patients. Beyond their insensitivity to radiotherapy or chemotherapy, we propose that surgical intervention can effectively remove tumors that are too large to be completely eradicated by radiotherapy, thereby positively impacting the prognosis.

The FIGO staging system is commonly employed to predict the prognosis of CCAC, with each stage typically exhibiting a strong correlation with overall survival. However, variations in outcomes among patients within the same stage suggest that factors such as age, marital status, and treatment strategies, which are not included in the FIGO criteria-based tumor staging, may significantly influence prognosis. In the current study, we developed a nomogram that integrates various variables, including demographic and clinicopathological factors, into a quantitative model. This model has been shown to outperform traditional staging methodologies, such as the American Joint Committee on Cancer (AJCC) and FIGO staging systems, in forecasting outcomes and guiding clinical decisions (10, 13, 32–34). Similarly, our results indicate that the nomogram demonstrates superior predictive capability and greater clinical benefit compared to the FIGO criteria-based tumor staging alone. This superiority was supported by the positive NRI and IDI of the nomogram versus the FIGO staging system, as well as the results from the decision curve analysis.

Patients were stratified into three risk groups based on their total points from the nomogram, and significant differences in overall survival among these groups were demonstrated using the Kaplan–Meier method and the Cox hazard ratio model. The nomogram showed improved discrimination among the three risk groups, particularly the high-risk group, compared to the FIGO staging system, as shown in Figure 2. Patients with total points > 191.67, who are characterized by unfavorable outcomes, require heightened attention.

The nomogram has demonstrated promising clinical utility. A strength of this analysis is the reduced selection bias as the sample used to construct the nomogram was extracted from 18 medical centers registered in SEER database. In line with the guidelines set forth by the Transparent Reporting of a Multivariable Prediction Model for Individual Prognosis or Diagnosis (TRIPOD) statement (35), we employed bootstrapping and cross-validation methods to calculate the C-index and generate calibration curves. In addition, a database excluding missing data served as a validation cohort, which also functioned as a sensitivity analysis. The promising results were effectively reproduced within the validation cohort. In summary, our nomogram may be a valuable tool for assessing the prognosis of patients with CCAC to date.

The present study has several limitations that warrant discussion. First, our model has undergone only internal validation, which is a common characteristic of many nomograms. Its effectiveness would benefit from external validation using a different cohort to further confirm its generalizability. Second, the data were collected retrospectively, leading to some missing information for certain variables. However, given the rarity of CCAC, conducting prospective randomized controlled studies presents significant challenges. To address this, missing data were imputed, and sensitivity analysis based on validation cohort demonstrated robust results. A potential limitation of using missForest package in R for imputing missing values is that if the missing data mechanism was dependent on unobserved variables, this imputation method could have introduced bias into the subsequent analyses. Third, our research covered the period from 2000 to 2021, and it is possible that some data may be outdated or missing. In addition, there may be variability in the research objects, methodologies, data sources, and other factors. Finally, the data available for cervical cancer patients in the SEER database lack important details, such as the exact location of positive lymph nodes, the exact chemotherapy and radiotherapy regimen, and the sequence of surgery and adjuvant therapies.

## Key messages

**What is already known on this topic**

Clear cell adenocarcinoma of the cervix (CCAC) is a rare malignancy with limited prognostic tools, and current risk stratification primarily relies on FIGO staging, which lacks treatment-specific considerations.

Existing survival models for CCAC are hindered by small sample sizes due to disease rarity, with no validated nomograms incorporating multimodal treatment data.

**What this study adds**

This study establishes the first population-based prognostic nomogram for CCAC using SEER data (*n*=630), integrating nine clinicopathological and treatment-related variables with robust validation through advanced statistical approaches (NRI, IDI, DCA).

We propose a novel dual-cohort validation framework combining machine learning-based data imputation (missForest) and complete-case analysis, addressing biases from missing values in rare cancer research.

The nomogram demonstrates superior discrimination (C-index >0.80) and clinical utility over FIGO staging, particularly in long-term (10-year) survival prediction.

**How this study might affect research, practice or policy**

**Clinical practice:** Provides a freely accessible tool to guide individualized survival prediction and adjuvant therapy decisions in CCAC, especially for patients with incomplete FIGO staging data.

**Research:** Sets a methodological precedent for leveraging SEER database and machine learning imputation in rare gynecologic cancer studies, facilitating future external validation through multi-institutional collaborations.

**Policy:** Highlights the need to update current CCAC management guidelines by incorporating treatment-responsive prognostic models beyond traditional staging systems.

## Conclusion

In conclusion, the nomogram in this study, which offers increased precision, robust clinical utility, and more accurate prognostic predictions compared to FIGO staging systems, can be employed to forecast the survival outcomes of patients with CCAC.

## Data Availability

Publicly available datasets were analyzed in this study. This data can be found here: the data used and analyzed during the current study are available from public databases, which were recorded detail in the Methods part. The software code used in this study can be acquired from the corresponding author on reasonable request.

## Footnotes

^1^ http://www.r-project.org/
