# Supplementary material for: Development of a predictive tool for long-term prognosis in clear cell adenocarcinoma of the cervix: a large population-based real-world study
Source: Front Med (Lausanne). 2025 Jun 20;12:1606685. doi: 10.3389/fmed.2025.1606685 (PMC12226462; doi:10.3389/fmed.2025.1606685)
Supplement: Supplementary file 1 [file Supplementary_file_1.docx]

**
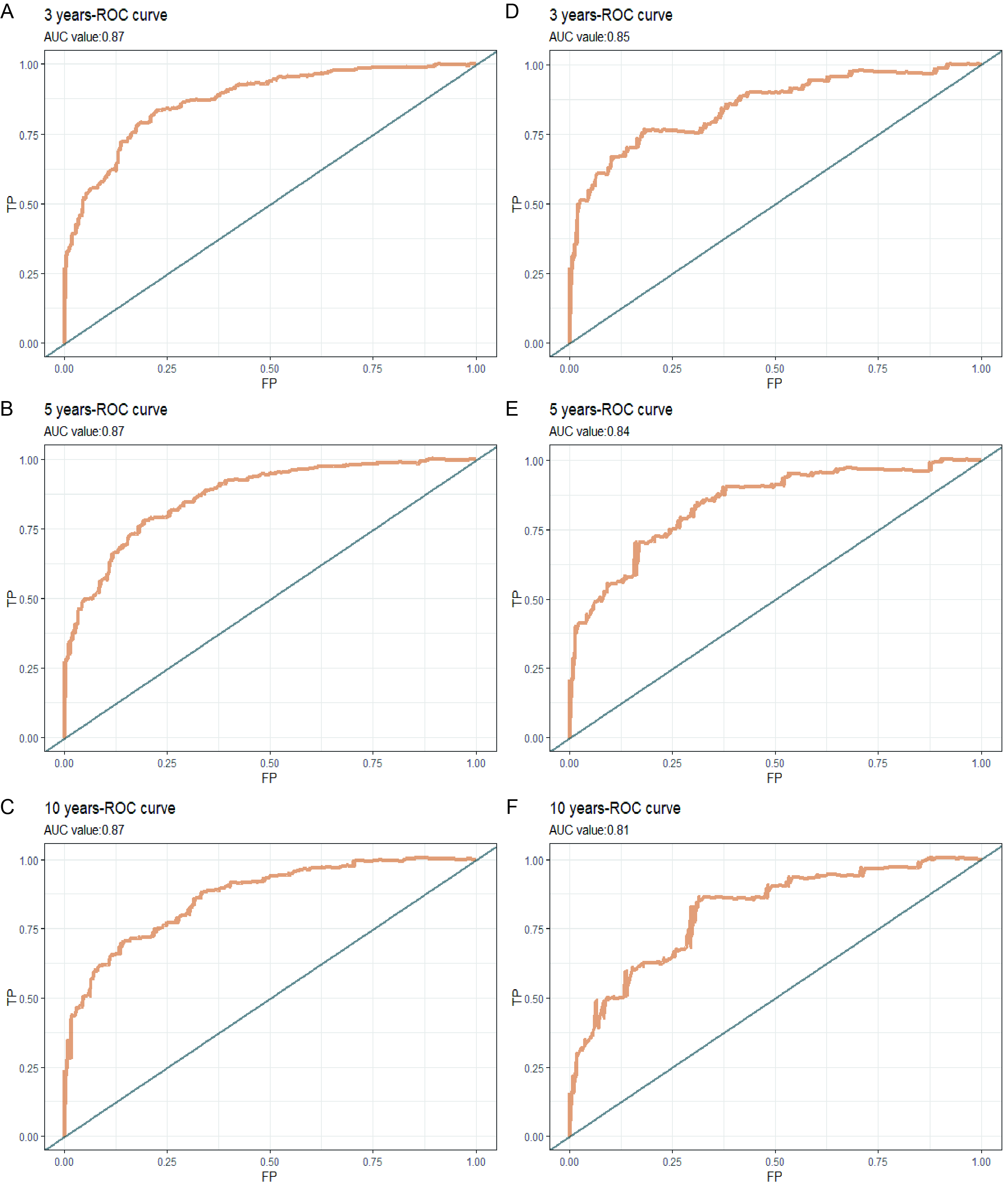
**

**Supplement Figure 1,** Receiver operating characteristic（ROC）curves for 3-, 5-, and 10-year OS in the training cohort **(A, B, C)** and validation cohort **(D, E, F)**

**
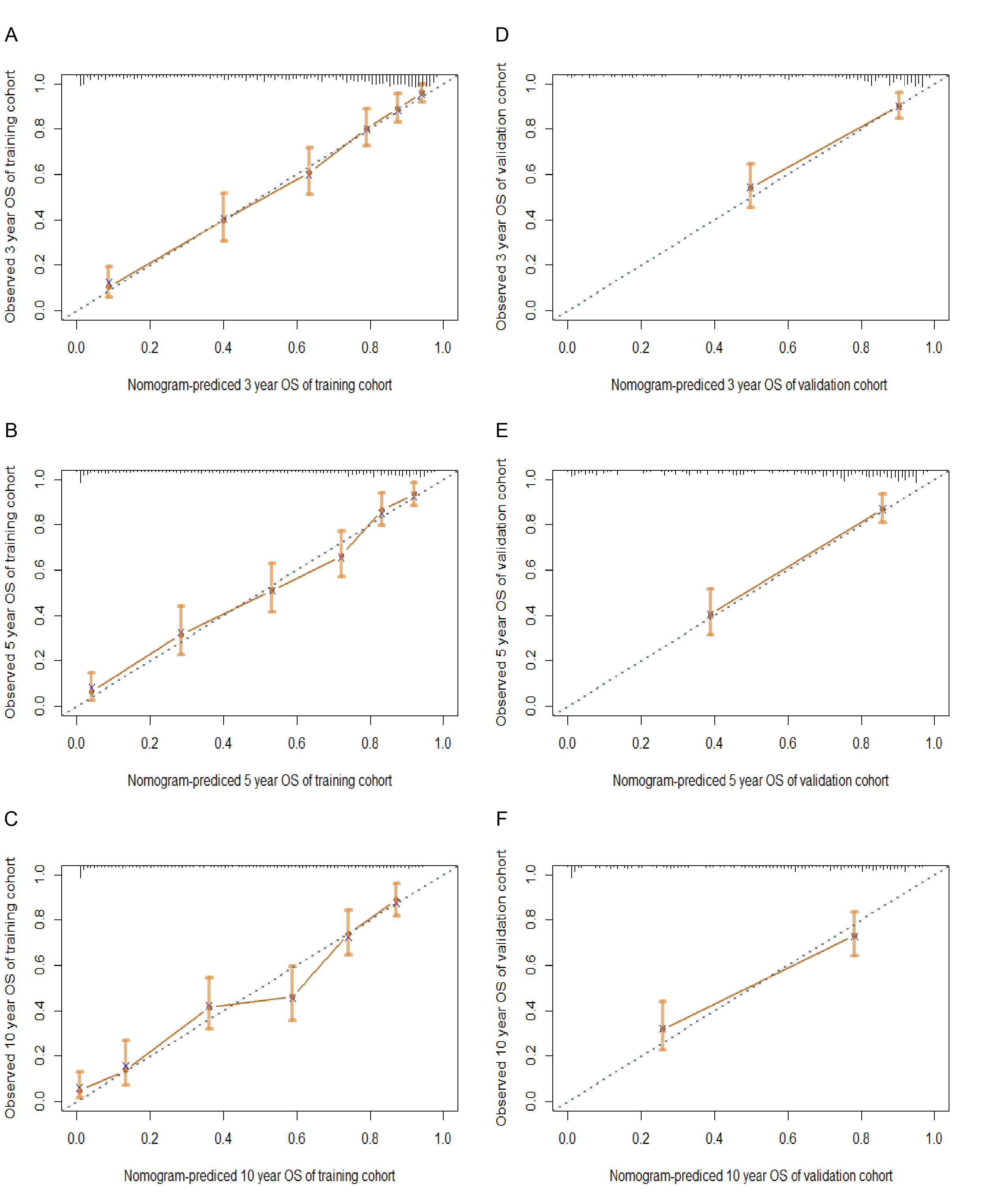
**

**Supplement Figure 2,** Calibration plots for 3-, 5-, and 10-year OS prediction for the training cohort **(A, B, C)** and validation cohort **(D, E, F)**.


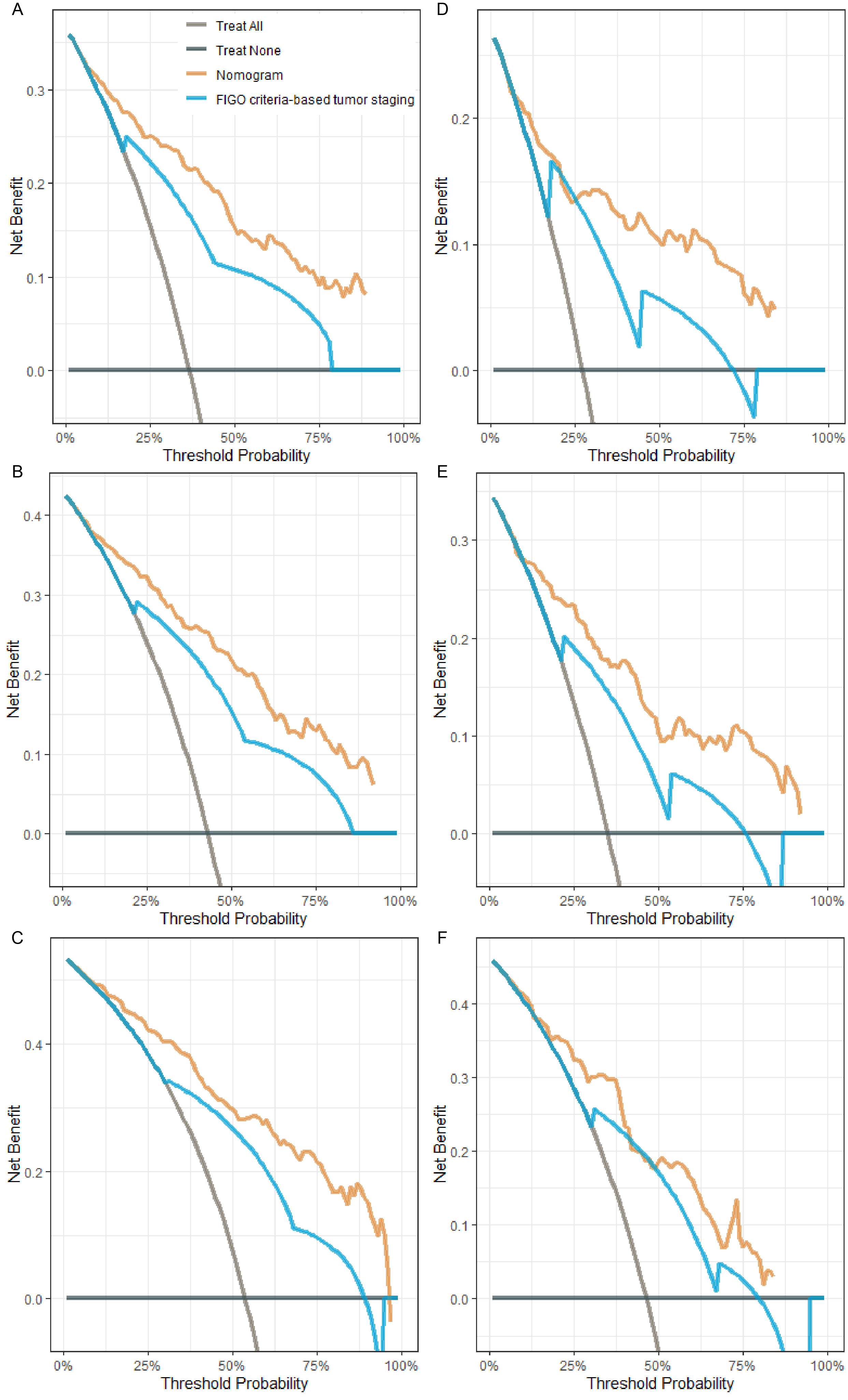


**Supplement Figure 3,** Decision curve analysis (DCA) of the nomogram and FIGO criteria-based tumor staging for predicting OS of patients with CCAC. **(A)** 3-year survival benefit in the training cohort. **(B)** 5-year survival benefit in the training cohort. **(C)** 10-year survival benefit in the training cohort. **(D)** 3-year survival benefit in the validation cohort. **(E)** 5-year survival benefit in the validation cohort. **(F)** 10-year survival benefit in the validation cohort. The x-axis represents the percentage of threshold probability, whereas the y-axis represents the net benefit, calculated by adding the true positives and subtracting the false positives. CCAC: clear cell adenocarcinoma of cervix; FIGO: The International Federation of Gynecology and Obstetrics.
